# Potential of plasmonics and nanoscale light–matter interactions for the next generation of optical neural interfaces

**DOI:** 10.1117/1.NPh.11.S1.S11513

**Published:** 2024-08-08

**Authors:** Filippo Pisano, Liam Collard, Di Zheng, Muhammad Fayyaz Kashif, Mohammadrahim Kazemzadeh, Antonio Balena, Linda Piscopo, Maria Samuela Andriani, Massimo De Vittorio, Ferruccio Pisanello

**Affiliations:** a Istituto Italiano di Tecnologia, Center for Biomolecular Nanotechnologies, Arnesano (Lecce), Italy; b University of Padua, Department of Physics and Astronomy “G. Galilei” Padua, Italy; c RAISE Ecosystem, Genova, Italy; d Università del Salento, Dipartimento di Ingegneria Dell’Innovazione, Lecce, Italy

**Keywords:** label-free optical neural interfaces, optical fibers, plasmonics

## Abstract

Within the realm of optical neural interfaces, the exploration of plasmonic resonances to interact with neural cells has captured increasing attention among the neuroscience community. The interplay of light with conduction electrons in nanometer-sized metallic nanostructures can induce plasmonic resonances, showcasing a versatile capability to both sense and trigger cellular events. We describe the perspective of generating propagating or localized surface plasmon polaritons on the tip of an optical neural implant, widening the possibility for neuroscience labs to explore the potential of plasmonic neural interfaces.

Over the past years, optical neural interfaces have gained widespread adoption in neuroscience laboratories, facilitating interdisciplinary collaborations and driving novel exploratory research. Neuroscientists teamed with physicists and engineers to develop photonic tools to improve light delivery and collection from scattering brain tissue, to generate microscopy and endoscopic methods to target hundreds of neurons simultaneously,^1^^,^^2^ or to image deep brain regions with high spatio-temporal resolution for monitoring neural activity^3^^,^^4^ or neurotransmitter release.^5^^,^^6^ Novel polymeric fibers,^7^^,^^8^ also employed for optical control of gut-brain interactions,^9^ have been devised in a synergistic effort with material scientists, and the interaction with chemists has enabled the use of colloidal up-conversion semiconductor nanoparticles to get point-like light sources close to the cell membrane.^10^^,^^11^

In this framework, the application of nanotechnology to neuroscience research holds the potential to revolutionize the way we interface with neural tissue with and without the use of genetically encoded optical actuators or neural activity indicators.^12^^,^^13^ Examples are the groundbreaking Neuropixel and SiNAPS technology,^14^^,^^15^ which exploit semiconductor nanofabrication to generate ultra-high-density neural recording implantable systems; the use of carbon nanotubes on flexible neural implants;^16^ and implantable aptamer field-effect transistors^17^ to perform electrochemical detection of neurotransmitters.

From a photonics perspective, integrated waveguides and phased arrays have been devised to obtain dynamically reconfigurable illumination patterns to control neural activity with spatio-temporal resolution^18^^,^^19^ or to select the wavelength sensed by implantable photodetectors with single-photon sensitivity.^20^ As an exploratory field, when scaled further down to the nanometer level, both dielectrics and noble metals can strongly enhance light–matter interactions, and the resulting physical phenomena can either sense or trigger cellular events^21^^–^^23^ (Fig. 1). In this framework, the coherent interaction between light and conduction electrons in metallic nanostructures is of particular interest because it can induce localized surface plasmon polariton (LSPP) in metallic nanoparticles (NPs) or surface plasmon polaritons (SPP) in periodic nanostructures. These plasmon resonances produce sub-wavelength localization of electromagnetic fields that generate enhanced absorption or scattering at the structure’s resonant wavelength.

**Fig. 1:**
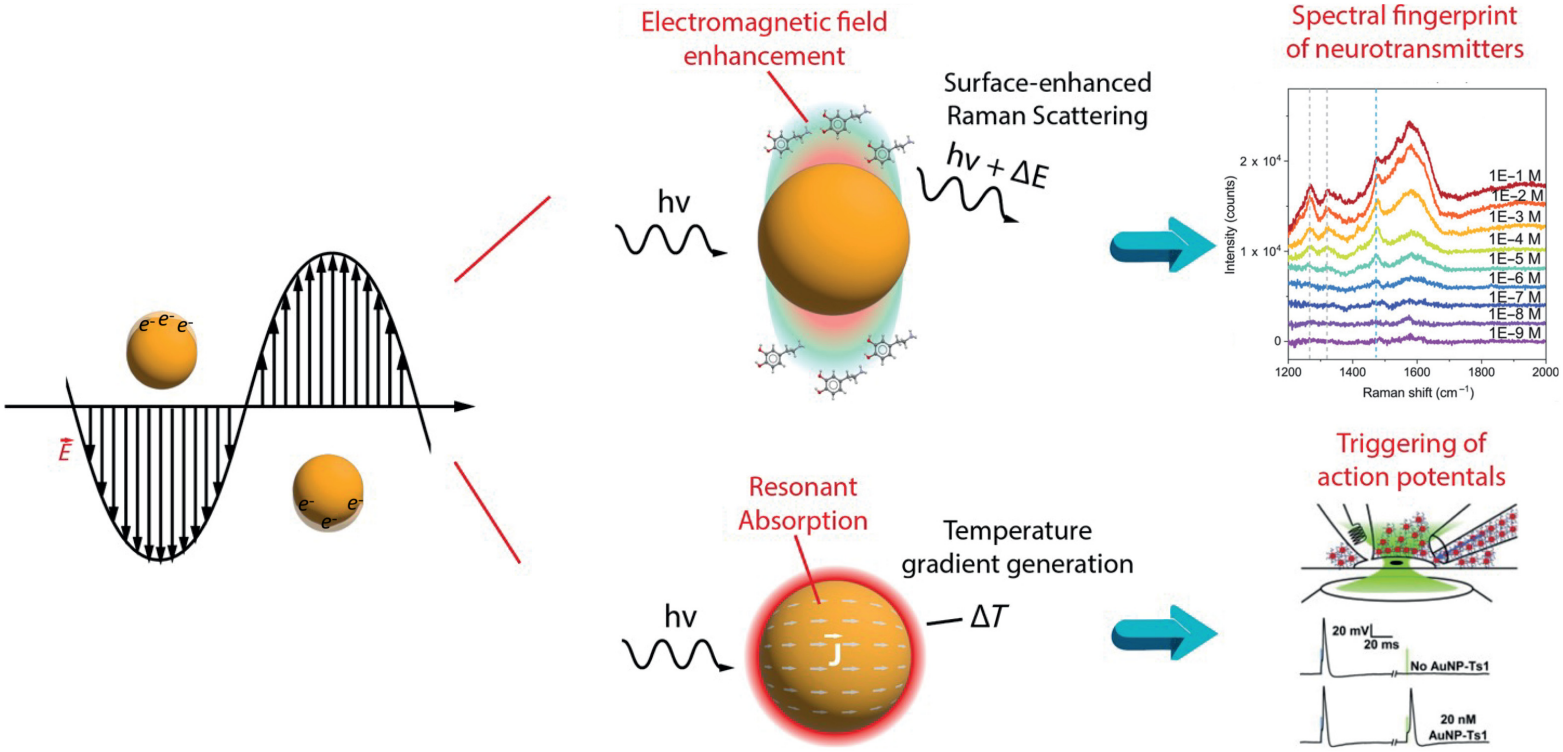
Surface plasmon resonances can be employed for high-sensitivity neurotransmitter detection through surface-enhanced Raman, as well as for triggering action potentials through local heat generation. The Raman spectrum on the top row is reproduced from Ref. 24. The panel titled “Triggering of action potentials” is reproduced from Ref. 21.

Both plasmonic-enhanced scattering and absorption have been deeply investigated for interfacing with neural cells *in vitro*. For example, surface-enhanced Raman scattering (SERS) can potentially enable neurochemical detection at attomolar concentrations through spectral measurements of photons scattered by the target molecule interacting with the plasmonic-enhanced electromagnetic field.^25^ Resonant absorption is instead at the base of thermoplasmonic heating (TPH), which exploits the enhanced light–matter interactions to provide a strong temperature gradient highly localized in space and time.^26^^,^^27^ When applied to nanoparticles conjugated with the cell membrane, TPH can modify the membrane capacitance locally and elicit action potentials.^21^ Optogenetics^28^ has revolutionized the ability to use light for precise control of neural activity within genetically targeted populations, and TPH has the potential to translate the complexity of genetic modification to less stringent requirements. Potential applications of TPH to control neural activity leverage nanoparticle-based light-heat mediators in proximity to cell membranes, instead of light-gated ion channels across the membrane.^21^ Despite TPH not being cell-type specific, NPs can be functionalized with appropriate ligands to target many different cell phenotypes,^21^ potentially enabling the capability to deliver plasmonic heat mediators to specific population of neurons. In addition, the nanoparticles’ LSPP resonances can still be excited at the diffraction limit, preserving the spatial resolution typical of optogenetic techniques.

As the most common applications of nanoplasmonics are exploited *in vitro* or are based on colloidal nanoparticle formulations injected *in vivo*, one of the next frontiers is represented by the possibility of generating plasmonic resonances on implantable photonic systems. This would allow for multiple benefits, notably (i) to facilitate applications of TPH stimulation and SERS sensing in deep brain regions, circumventing the limitations imposed by tissue scattering by enabling the immediate interaction of guided photons with the plasmonic structures; (ii) to avoid the direct injection of nanoparticles into the central nervous system, the fate and toxicity of which are still under debate;^29^^,^^30^ and (iii) to provide a remote and dynamic control on the excitation field to optimize the exploitation of plasmonic sensing.

For this to be effective, several conditions should coexist on the same neural probe. First, the light-guiding properties of the probe should allow for exciting the plasmonic resonances, for example, enabling momentum-matching in periodic nanostructures supporting SPPs.^31^ Then, the nanostructures should be distributed on a surface wide and thin enough to extend the plasmonic enhancements from the nanoscale to a physiological scale (tens or hundreds of micrometers), matching with the size of the cellular group of interest. Finally, tight adhesion of the nanostructures on the probe is also a key requirement to prevent nanotoxicity that, for example, can be caused by the bioaccumulation of NPs, which can be harmful independently from the reactivity of their material in a bulk formulation.^32^

Considering these requirements, multimodal optical fibers (MMFs) are a promising candidate for developing implantable neuro-plasmonic systems^22^^,^^24^^,^^31^^,^^33^ (Fig. 2). The wealth of information that can be transferred through several hundreds of modes is accompanied by a relatively high numerical aperture (NA) compacted in an implant cross-section that can range from a few hundreds down to a few micrometers. The NA is directly linked to the maximum transversal component of the wavevector ($k_{T}$) of guided light, which is the main parameter affecting the momentum-matching condition. Control over the $k_{T}$ (within the limit imposed by NA) can be gained by employing phase modulation at the input of the waveguide^33^^,^^34^ [Fig. 2(a)], by injecting light at a well-defined input angle,^35^ or by tapering the waveguide^36^ [Fig. 2(b)]. Peculiarly, this control can be obtained over the entire fiber facet or in a reconfigurable sub-region of the plasmonic surface.^33^ For fabrication, plasmonic structures can be integrated onto silica-based MMFs with either nano-patterning a previously deposited material (the so-called *top-down* fabrication) or direct nucleation of the nanostructures directly on the implantable waveguide (usually referred to as *bottom-up* or *self-assembly*). Top-down methods are better suited for controlled patterning of small resonators, whereas bottom-up approaches benefit from low costs and high throughput, making them more appropriate for wide surface structuring. Gold NPs can be deposited on either flat-cleaved or tapered optical fibers by bottom-up methods including electrostatic self-assembly, dip coating, laser-assisted evaporation, or solid-state dewetting, with this latter providing for ligand-free, highly dense, and statistically uniform coverage of NPs^24^ [Fig. 2(c)]. Depositing nanometer-thin dielectric films on top of the nanostructures or introducing adhesion metal layers (for instance with Ti) during the fabrication process can further engineer the NP adhesion properties on optical fiber, mitigating potential release in brain tissue. In addition to silica-based MMFs, counting for high transmission and minimal autofluorescence across visible and infrared spectra, soft polymeric fibers are also emerging as promising candidates for applications in plasmonic neural interfaces. Recent advancements have successfully induced SPPs within both continuous and patterned gold thin films hosted on polymeric fibers, complementing the distinctive mechanical attributes of these fiber optics and paving the way for innovative sensing methodologies.^37^^,^^38^

**Fig. 2:**
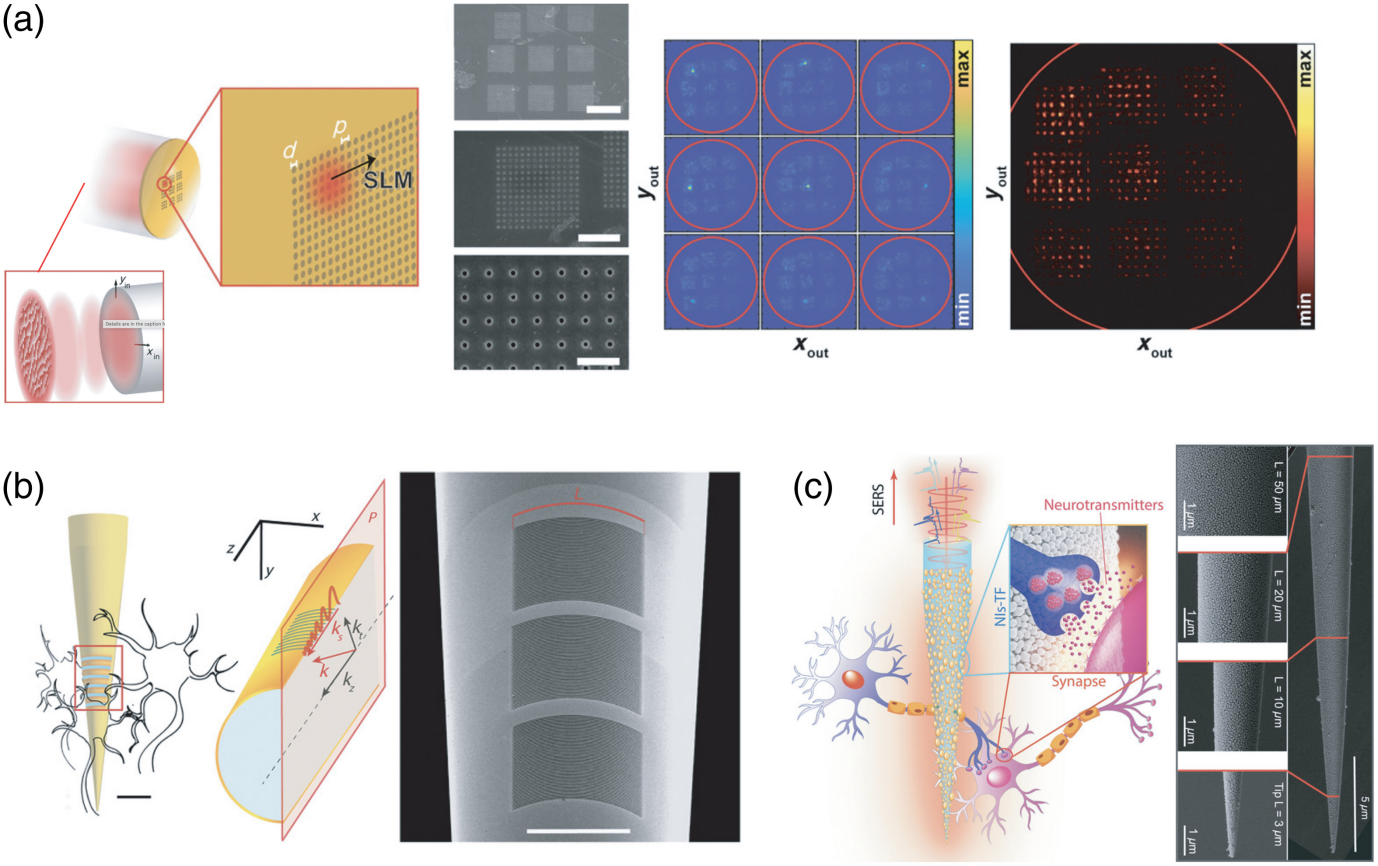
(a) Wavefront shaping can be employed to obtain the momentum-matching condition on the nanostructured facet of a multimode optical fiber. Reproduced from Ref. 33. (b) Implantable tapered optical fibers hosting plasmonic nanogratings. Reproduced from Ref. 31 (c) Plasmonic gold nanoislands nucleated directly onto the taper of an implantable fiber. Reproduced from Ref. 24.

Therefore, the combination of waveguide optics, nanofabrication, and light–matter interactions has the potential to gather the neurophotonics and neuroscience community to explore the use of *in vivo* neuroplasmonics. Although waveguide-based neuroplasmonics remains in its nascent stages, its potential applications are vast, particularly in targeting deep brain regions or structures spanning significant dorso-ventral extents. The ability to manipulate the near field properties through either SPPs or LSPPs can indeed enable the exploration of spatially resolved refractive index sensing to study the local optical properties of brain tissue,^39^^,^^40^ SERS imaging to monitor neurochemicals dynamics at very low concentrations,^24^^,^^25^^,^^33^^,^^41^ and the implementation of nonlinear optical methods such as surface-enhanced coherent anti-stoke Raman imaging.^42^ Moreover, prospective applications also include the realm of genetically encoded fluorescent indicators of neural activity. For instance, leveraging the far-field response of periodic plasmonic structures enables the generation of scattering-resilient Bessel beams,^43^^,^^44^ thereby potentially enhancing functional fluorescence excitation. Furthermore, exploiting the relationship between resonant wavelength, wavevector, and guided modes offers opportunities for novel multiplexing methods,^31^ enriching the breadth of information extractable from the brain through a single neural interface.

In conclusion, we view plasmonic neural interfaces as a promising research direction to complement the existing approaches for neural interfaces with the central nervous system. Interestingly, functionalities such as TPH and SERS sensing might be integrated alongside the existing approaches based on optical, photonic, and high-density opto-electrical architectures, synergistically adding one more technique to the available tools for studying the central nervous system.

## Data Availability

Data sharing is not applicable to this article, as no new data were created or analyzed.
